# Octreotide and hepatocellular carcinoma

**DOI:** 10.1038/sj.bjc.6603799

**Published:** 2007-05-15

**Authors:** F Farinati, A Sergio, A Baldan, P Zucchetta, V D Corleto

**Affiliations:** 1 Department of Surgical and Gastroenterological Sciences, Venetian Institute of Oncology (IOV), Padua University, Padua, Italy; 2 Department of Diagnostic Medical Sciences, Padua University, Padua, Italy; 3 Department of Digestive and Liver Diseases, II School of Medicine, University ‘La Sapienza’, Rome, Italy

**Sir**,

The treatment of advanced hepatocellular carcinoma (HCC) in patients no longer eligible for surgical, percutaneous or transarterial therapies is dismal. Chemotherapy, anti-estrogens or anti-androgens, retinol derivatives or stem cell factors have been tested with poor results, and we eagerly wait for the results of ‘biologic’ treatments, such as sorafenib.

Since the first enthusiastic report on Octreotide (Kouroumalis *et al*, 1998), in which HCC patients were characterized by somatostatin receptor expression and survival was significantly longer in the octreotide-treated arm, several papers have been published that almost constantly failed in confirming these preliminary data (Yuen *et al*, 2002; Slijkhuis *et al*, 2005).

The recent papers by Cebon in the British Journal of Cancer (Cebon *et al*, 2006) and by Becker in Hepatology (Becker *et al*, 2007) are a kind of obituary to the use of the drug in HCC and will probably lead to a definite stop to any attempt to treat HCC with octreotide, which is also an expensive drug. Indeed, in the two studies not only had the drug no impact on survival, even the quality of life was absolutely unaffected by the treatment. In the former study, only about 50% of the patients had a positive Octreoscan, while in the latter, the receptor status was not assessed. One could therefore say that the chances for the drug to work in receptor-negative patients are quite small. In previous papers, the percentage of receptor-positive patients was not defined (Treiber *et al*, 2006) or showed wide variability (Reynaert *et al*, 2004), even in relation to the type of receptor tested (Blaker *et al*, 2004).

In Italy, the rules for prescription require a positive Octreoscan as mandatory and we therefore recruited 25 consecutive patients diagnosed in the last 6 months, who had advanced stage HCC, according to the American Association for the Study of Liver Disease (AASLD) (Bruix and Sherman, 2005) (Table 1) and performed the imaging technique. In contrast with that found by Cebon, only 2/25 (8%) patients tested positive. Both had multifocal disease, hepatatis C virus-related aetiology, one had a large size HCC (Figure 1) and very high α-fetoprotein levels (245.000 *μ*gdl^−1^). One of the two refused the treatment. The second soon became intolerant, developed diarrhoea and nausea, interrupted the treatment after 2 months and died of upper gastrointestinal bleeding at 4 months.

As in our experience, the somatostatin receptor (SSTR) expression in HCC is independent of tumour stage, differentiation, underlying liver disease and/or histological type (Blaker *et al*, 2004) and none of these parameters is therefore predictive of somatostatin analogue treatment response. As said, selection of HCC patients with more sensitive methods of SSTR expression (reverse transcription – polymerase chain reaction or immunohistochemistry, among which a high correlation is present), have not and will not change the picture. The clinical availability of new synthetic SSTRs pan-inhibitors such as SOM230 or BIM-23A779 (high affinity for SSTR1, SSTR2, SSTR3, SSTR5), could define whether the low or absent of *in vivo* effect on proliferation and apoptosis of the available analogues is related to the incapacity to stimulate a specific SSTR subtype or to a generally modest antiproliferative activity of these receptors (Reynaert *et al*, 2004).

## Figures and Tables

**Figure 1:**
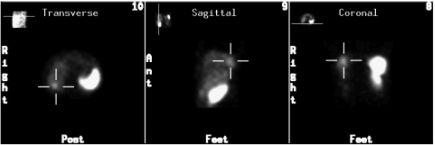
Octreotide Scintigraphy showing pathologic uptake in right liver lobe and normal uptake in spleen.

**Table 1:** Clinical features of the HCC patients recruited

**Variables**	***N* (total 25)**	**%**
*Gender*		
Male	20	80
Female	5	20
		
*Aetiology disease*		
HBV	1	4
HCV	16	65
Alcohol (+/− viruses)	8	31
		
*AFP*		
<20 ngdl^−1^	8	31
20–200*μ*gl^−1^	10	42
>200 *μ*gl^−1^	7	27
		
*Lesion number*		
1–3	7	29
>3	18	71
		
*Tumour size*		
<5 cm	19	74
>5 cm	6	26
		
*Edmonson's grade of differentiation(in patients biopsied)*		
Well differentiated	5	71
Moderately differentiated	2	29

Abbreviations: AFP, α-fetoprotein; HBV, hepatitis B virus; HCV, hepatitis C virus.
